# Targeting collagen to optimize cancer immunotherapy

**DOI:** 10.1186/s40164-025-00691-y

**Published:** 2025-07-29

**Authors:** Yida Wang, Feng Zhang, Zhiwen Qian, Ying Jiang, Danping Wu, Lu Liu, Xin Ning, Jie Mei, Daozhen Chen, Yan Zhang

**Affiliations:** 1 Department of Oncology, Wuxi Maternal and Child Health Care Hospital, Wuxi Medical Center, Nanjing Medical University, Wuxi, 214002 China; 2 Department of Oncology, Wuxi Maternity and Child Health Care Hospital, Women’s Hospital of Jiangnan University, Jiangnan University, Wuxi, 214002 China; 3 The First Clinical Medicine College, Nanjing Medical University, Nanjing, China

**Keywords:** Collagen, Biomarker, Immunotherapy, Tumor microenvironment, Cancer, Clinical applications

## Abstract

Collagen in the tumor microenvironment plays diverse biological roles, from serving as the structural framework of tumors to influencing immune responses, angiogenesis, and tumor progression. Consequently, developing strategies to optimize the suppression of collagen’s promotive effects on tumor growth while maintaining its inhibitory functions on tumor initiation has become a key focus of cancer research and therapy. A significant challenge remains in identifying a biomarker with both high sensitivity and specificity for cancer diagnosis. This review, therefore, highlights the substantial value and clinical relevance of collagen as a biomarker throughout cancer onset and progression. It explores the fundamental link between collagen and immunotherapeutic outcomes, further illustrating how targeting collagen—along with its interactions with tumors and immune cells—can offer more reliable predictive markers for personalized immunotherapy. This approach ultimately enables the development of more tailored and standardized treatment regimens for patients with cancer.

## Graphical Abstract

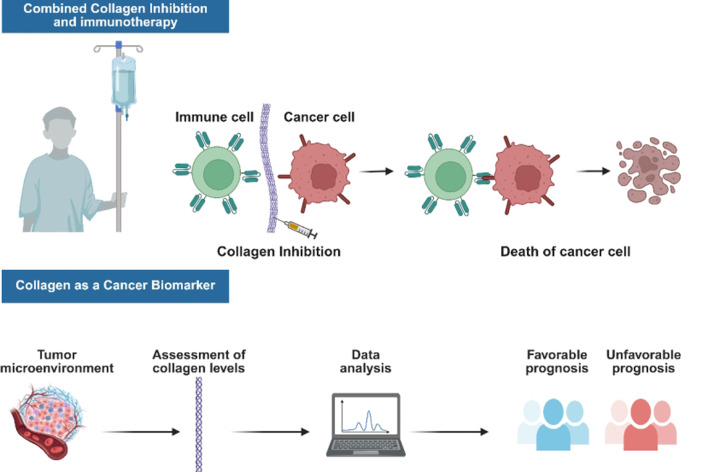

## Background

Cancer ranks as the second leading cause of death both in the United States and globally. In 2024, an estimated 2,001,140 new cancer cases and 611,720 cancer-related deaths are projected in the U.S. alone, placing cancer second only to heart disease in terms of mortality [1]. Lung, colorectal, and pancreatic cancers are among those with the highest fatality rates [1]. By 2040, the global cancer burden could reach 28.4 million cases, marking a 47% increase compared to 2020 [2]. Over recent decades, two major breakthroughs in cancer therapy have emerged: the targeting of oncogene-driven tumors through molecular targeted therapy and advancements in tumor immunology, such as immunotherapy [3, 4]. Immunotherapy, which leverages the host immune system to either passively or actively combat malignancies, has garnered significant attention. Notable approaches, including immune checkpoint blockade (ICB)—which enhances immune responses against cancer cells by inhibiting specific proteins on tumor or immune cell surfaces—cancer vaccines, and adoptive cell transfer, which genetically modifies a patient’s immune cells to recognize specific tumor antigens, show immense promise as transformative cancer treatments [5].

With the rise of immunotherapy, a new paradigm in cancer treatment has emerged, shifting focus from solely cancer cells to the broader tumor microenvironment (TME), a critical determinant in tumor formation, progression, and metastasis. The extracellular matrix (ECM), a pivotal component of the TME, is predominantly secreted by cancer-associated fibroblasts (CAFs), a heterogeneous and plastic subset of activated fibroblasts. Collagen, a primary ECM component, plays a pivotal role in the structural organization of solid tumors, influencing tumor invasion, growth, and metastasis [6]. Its abundance and arrangement are intimately associated with tumor behavior. Numerous studies have demonstrated the upregulation of various collagen types across diverse malignancies, correlating closely with the efficacy of immunotherapeutic treatments [7, 8]. This review focuses on collagen, examining its essential properties and its role within the tumor immune microenvironment. Special attention is given to its potential as a biomarker for cancer initiation and progression and its synergistic effects with immunotherapy in clinical settings. These findings may provide novel insights into cancer monitoring, diagnosis, and therapeutic strategies.

## Collagen fundamentals

### Collagen structure

Collagen is one of the most prevalent proteins in the human body, representing approximately one-third of the total protein content [9]. Predominantly found within the ECM, collagen members also contribute to cell membranes. In addition to their critical role in determining tissue architecture and morphology, they are involved in cellular adhesion, proliferation, and migration [2]. Collagen consists of three polypeptide chains that form a stable triple helix. The amino acid composition of these chains, typically including glycine, proline, and hydroxyproline, contributes to their remarkable thermal stability. The α-chains undergo enzymatic processing through N-terminal and C-terminal propeptide regions to form mature, triple-helical collagen. Although stable in neutral environments, this triple helix loses stability in charged conditions. Lysyl oxidase (LOX) catalyzes crosslinking between collagen molecules, resulting in the formation of collagen fibrils or networks [10].

The collagen superfamily consists of 28 distinct members, classified into fibrillar and non-fibrillar types [11, 12]. These are further categorized based on structure, localization, and biological function, encompassing fibril-forming collagens, fibril-associated collagens with interrupted triple helices, basement membrane collagens, beaded filament collagens, short- and long-chain collagens, collagens with multiple triple-helix domains, and membrane-associated collagens with interrupted triple helices. Collectively, these subtypes contribute to a complex matrix that provides structural integrity to tissues. Among them, Collagen type I (COL I), Collagen type III (COL III), and Collagen type V (COL V) are primarily synthesized by fibroblasts, while Collagen type IV (COL IV) is predominantly expressed by epithelial and endothelial cells [13]. Research has predominantly focused on these subtypes. COL I, the most abundant, consists of two α1 chains and one α2 chain. In skin, it accounts for 80–85% of the dermal ECM, and in bone tissue, it exceeds 90% ^8^.

### Collagen function

Under normal physiological conditions, particularly during skin wound healing, collagen, together with elastin, ranks among the most abundant proteins in the skin [14]. Upon skin injury, platelets interact with exposed collagen from the ECM and endothelium, triggering coagulation and the clotting cascade [15]. During the repair process, changes in collagen subtypes, quantities, and organization directly influence the tensile strength of scar tissue. Collagen type XVII (COL XVII) plays a pivotal role in wound healing, acting as a stem cell marker and reflecting their self-renewal capacity, thus positioning it as a promising therapeutic target for anti-aging and wound treatments [16]. Beyond its structural contribution to wound healing, collagen engages with other ECM components and cytokines to modulate inflammatory and reparative responses, stimulating the secretion of inflammatory cytokines and growth factors by surrounding cells [17]. In certain tissue regeneration contexts, collagen serves as a guiding scaffold; for instance, in bone repair, extracellular mineralization of collagen mimics osteogenic processes, allowing osteoblasts to secrete and mineralize bone matrix on collagen scaffolds [18].

Beyond its roles in wound healing, tissue regeneration, and structural support, collagen—primarily structural types—also impacts cell signaling, adhesion, proliferation, metastasis, and immune cell recruitment [19]. As the key structural component of all connective tissues, collagen is omnipresent in parenchymal organs, providing mechanical rigidity and structural stability. Its presence in basement membranes is closely associated with angiogenesis and cellular survival. Notably, COL IV within vascular endothelial cell (VEC) basement membranes plays a pivotal role in regulating angiogenesis [20]. By interacting with ECM components such as laminin, COL IV creates a stable structural interface that not only offers mechanical support but also acts as a permeability barrier, maintaining homeostasis between the blood vessel interior and exterior. Additionally, RAS family proteins (RAB10 and RAB25) regulate COL IV through the transport of lysyl hydroxylase 3, ensuring vascular stability [21]. Consequently, a comprehensive investigation of collagen’s role in vascular remodeling could inform the development of angiogenesis-targeted therapies in oncology.

Collagen not only serves as the structural foundation of tissue architecture but also plays a pivotal role in remodeling processes that drive TME progression, particularly in tumor cell growth, infiltration, and metastasis. For example, collagen type X A1 facilitates epithelial–mesenchymal transition (EMT), promoting tumor cell dissemination. COL I shows potential as a diagnostic marker for metastatic tumors and is implicated in both primary bone cancers and cancer-associated bone metastases [22]. CAFs are the primary source of collagen synthesis within the TME, producing ECM components and regulating the expression of organ-specific and immunomodulatory chemokines [23]. In addition to CAFs, keratinocytes, VECs, smooth muscle cells, macrophages, and cancer cells also contribute to collagen synthesis.

Numerous receptors on the cell surface recognize collagen, including integrins, discoidin domain receptors (DDRs), glycoprotein VI, the osteoclast-associated receptor, and G-protein-coupled receptor 56 [24–26]. These collagen–receptor interactions establish essential adhesion sites that facilitate signal transduction, proliferation, metastasis, and immune cell recruitment in tumor cells.

**Fig. 1:**
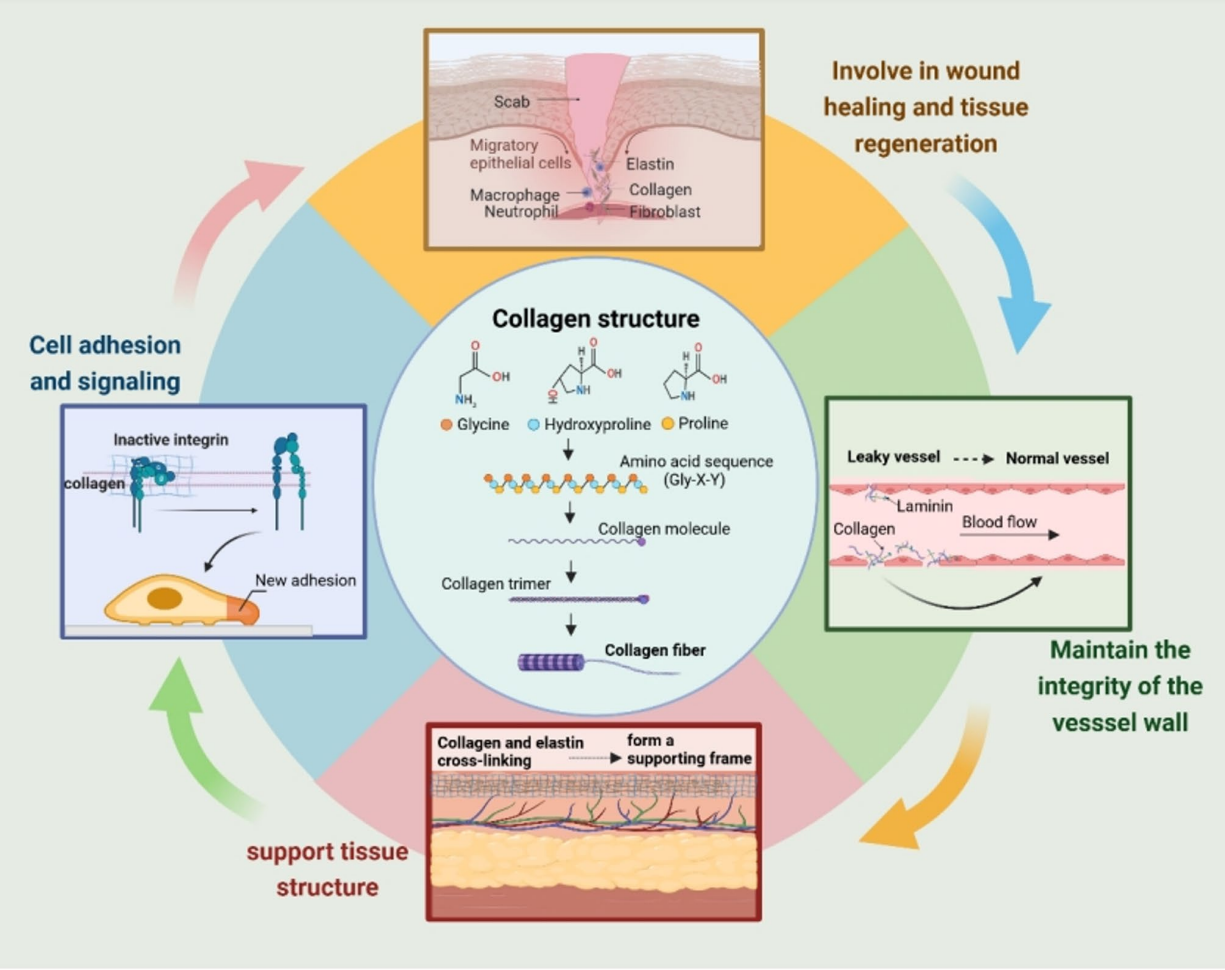
Structure of collagen and its physiological function. Collagen is characterized by three polypeptide chains that intertwine to form a helical structure. These chains typically follow a Gly–X–Y sequence (glycine, proline, and hydroxyproline) and can be processed into a mature triple helix through enzymatic activity. Under normal physiological conditions, collagen plays a critical role in wound healing and tissue remodeling by influencing cell adhesion, signal transduction, supporting tissue architecture, and maintaining the structural integrity of vascular walls. Created in BioRender.com

In summary, collagen constitutes a diverse and functionally critical protein family, influencing various physiological processes, from tissue regeneration and wound repair to cell migration, angiogenesis, and the shaping of the TME. This complexity emphasizes its substantial research potential in tumor immunology and regenerative medicine. Following the exploration of collagen’s core properties, its dynamic behavior within the TME and its impact on tumorigenesis and progression are further analyzed (Fig. 1).

### Collagen synthesis and degradation

Collagen biosynthesis is a multifaceted process involving both intracellular and extracellular stages [2]. It initiates in the cell nucleus, where mRNA transcripts for α-chains are synthesized, before migrating to the rough endoplasmic reticulum (RER) for translation into prepro-α-chains. During this process, signal peptides are cleaved from the N-terminus, generating pro-α-chains, which undergo essential post-translational modifications, including hydroxylation and glycosylation, to ensure collagen stability and function. These modified pro-α-chains then assemble into a procollagen monomer, linked by disulfide bonds at the N- and C-terminal propeptide regions, forming a triple helix. This monomer is further modified with oligosaccharides in the Golgi apparatus before being packaged into secretory vesicles for extracellular export. Once outside the cell, collagen peptidases cleave the propeptide regions, converting procollagen to tropocollagen, which spontaneously assembles into fibrils. LOX facilitates the formation of covalent crosslinks between collagen molecules, stabilizing the fibril structure. CAFs play a significant role in modulating collagen’s physical properties through LOX production. The dynamic balance of the ECM is maintained by collagenases and cathepsins that degrade collagen molecules.

Collagen degradation also involves multiple enzymes and pathways, reflecting the protein’s tightly coiled triple-helical structure, which offers substantial resistance to proteolysis. Only a few proteases are capable of cleaving native type I collagen under physiological conditions, primarily matrix metalloproteinases (MMPs) and certain cysteine proteases. The collagenases of the MMP family, including MMP-1, MMP-2, MMP-8, MMP-13, and MMP-14, are the main enzymes responsible for collagen digestion [27].

**Fig. 2:**
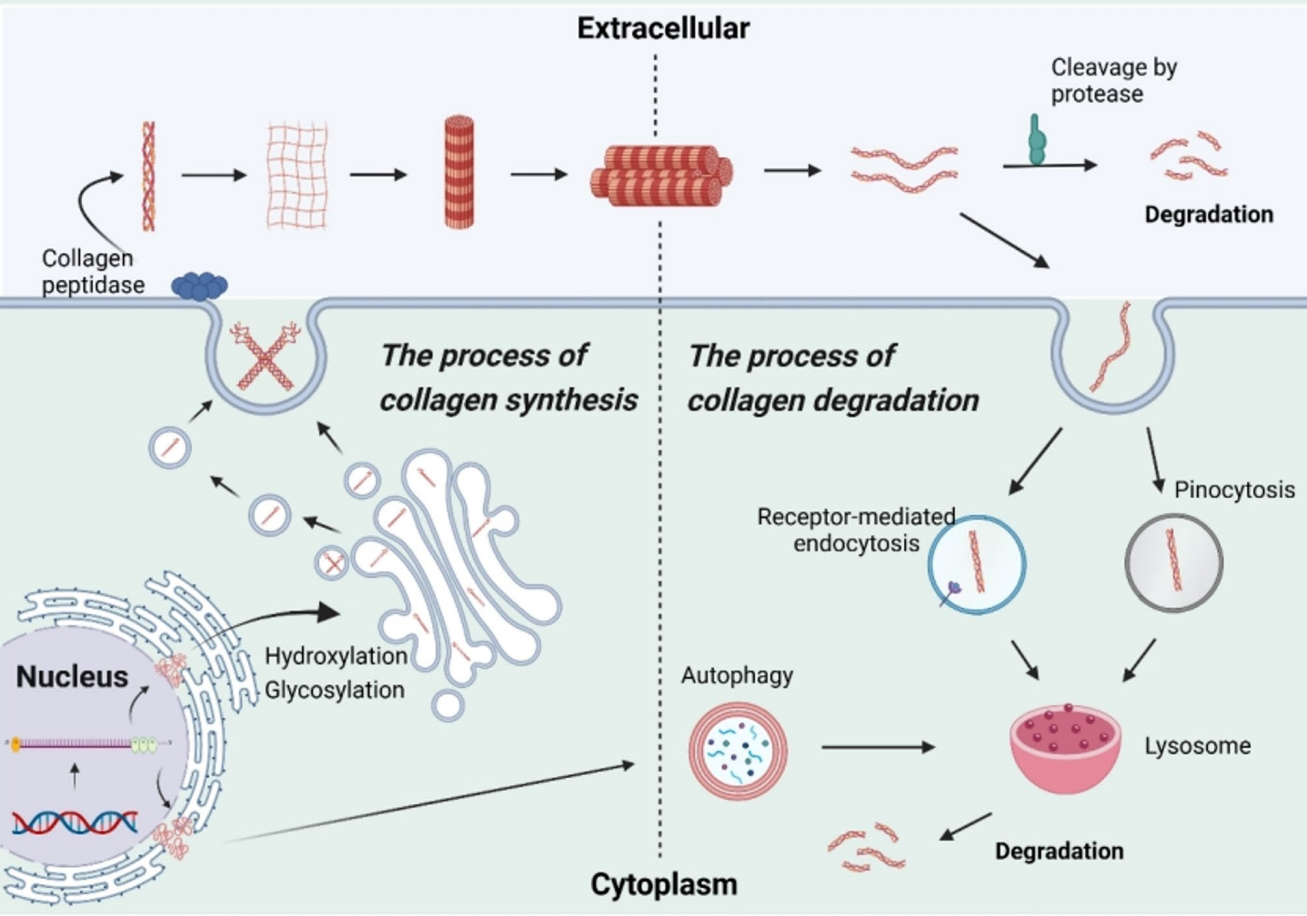
Synthesis and degradation of collagen. Collagen synthesis begins with the transcription of the α-chain gene into mRNA in the cell nucleus, followed by its migration to the rough endoplasmic reticulum (RER), where it is translated into a pre-α chain. The N-terminal signal peptide is cleaved from the pre-α chain, which is then subjected to post-translational modifications in the RER lumen, including hydroxylation and glycosylation. Three modified pre-α chains assemble into a procollagen monomer, stabilized by disulfide bonds at both the N- and C-termini, forming a triple-helical structure. Further modifications, such as the addition of oligoglycans, occur in the Golgi apparatus. The procollagen is then packaged into secretory vesicles for extracellular release and converted to collagen by collagen peptidases that remove the propeptide regions. The resulting monomers self-assemble into fibrils, which aggregate into collagen fibers. Collagen degradation occurs through three primary pathways: (i) intracellular degradation *via* lysosomes; (ii) extracellular cleavage by soluble and membrane-bound proteases (e.g., MMPs); and (iii) internalization *via* pinocytosis or receptor-mediated endocytosis, followed by lysosomal degradation. Created in BioRender.com

In healthy tissues, ECM remodeling—primarily driven by collagen degradation—occurs in cycles of synthesis, secretion, and degradation [28]. This process can proceed through (i) lysosomal degradation of newly synthesized collagen, (ii) extracellular proteolysis mediated by soluble and membrane-bound proteases (e.g., specific MMPs), which target different cleavage sites across collagen subtypes, and (iii) endocytosis, wherein collagen is internalized *via* macropinocytosis or receptor-mediated uptake and subsequently degraded in lysosomes (Fig. 2).

MMPs play a critical role in degrading collagen fibers, proteoglycans, and other ECM components, thereby compromising tissue integrity and structural stability while facilitating cancer cell migration and dissemination [29]. For example, MMP-1 and MMP-13 are key contributors to tumor invasion and metastasis. MMP activity is regulated by several factors, including transcriptional control, activation of precursor enzymes, and inhibition by specific inhibitors such as TIMPs [30]. These regulatory mechanisms maintain a dynamic equilibrium in collagen degradation, balancing physiological and pathological needs.

### Role of collagen in the tumor microenvironment

#### Collagen and tumor cells

**Fig. 3:**
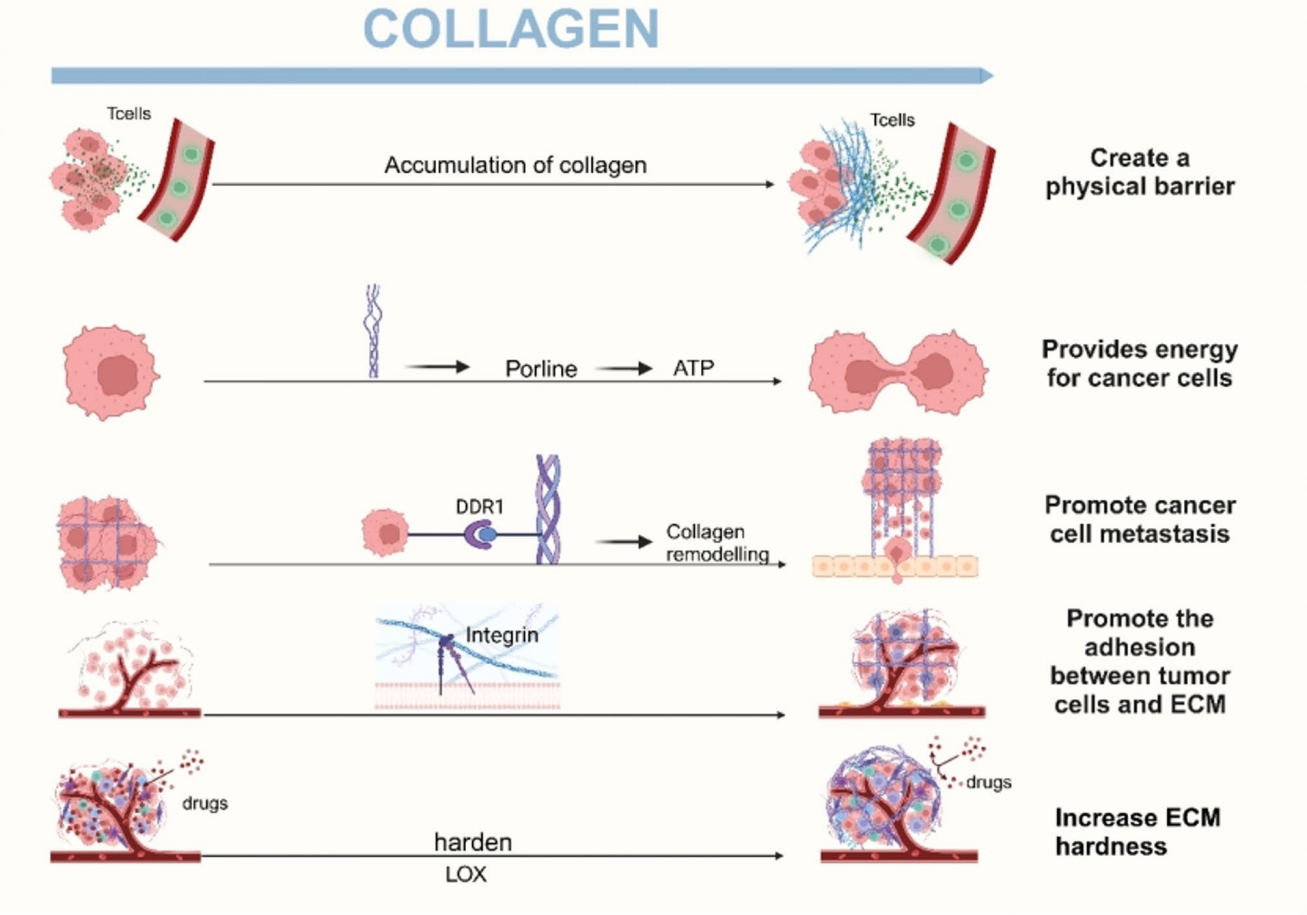
Function of collagen in the TME. In the TME, collagen accumulation forms a physical barrier, restricting tumor cell spread. Elevated collagen density increases matrix stiffness, which may enhance tumor cell invasiveness. Collagen also serves as a key energy source, supplying metabolic fuel for tumor growth. Additionally, tumor cells can induce collagen remodeling within the ECM, with the remodeled collagen environment subsequently influencing tumor cell behavior. Created in BioRender.com

Collagen exerts a multifaceted influence on tumor cell growth, proliferation, infiltration, and metastasis, with tumor cells often migrating along collagen fibers. Such interactions can activate a variety of signaling pathways (Fig. 3). Increased collagen density may induce morphological and functional changes in tumor cells, enhancing their invasiveness [27]. Additionally, collagen accumulation can create physical barriers that hinder tumor cell spread, although MMPs secreted by tumors can degrade ECM components, facilitating metastasis [11].

Collagen serves as a key energy source for tumor cell growth. For example, COL I provides metabolically available substrates indirectly through byproducts generated by collagen-degrading stromal cells. The breakdown of collagen can also promote tumor growth, with proline, a degradation product, supporting tumor progression [31]. Inhibiting collagen uptake by tumor cells or using insoluble collagen can induce tumor shrinkage [28]. Additionally, collagen modulates the adhesion between tumor cells and the surrounding stroma through interactions with integrin family adhesion molecules. COL I and COL IV, in particular, significantly influence tumor progression by binding to these adhesion receptors. Studies show that other ECM components also play essential roles in collagen synthesis and deposition. For instance, the glycoprotein fibronectin, which functions in the extracellular space, affects both collagen deposition and adhesion. Moreover, research has demonstrated that fibronectin’s interaction with focal adhesions can alter its conformation, exposing binding sites for other ECM molecules. As a result, fibronectin binds to collagen, laminin, and other proteins, facilitating cell adhesion to the ECM and promoting migration [32] (Tables 1, 2 and 3).

Various cell-surface receptors recognize collagen, and upon binding, directly regulate cancer cell metabolism, proliferation, and malignancy. Integrins, consisting of α and β subunits, are the primary receptors mediating tumor–collagen interactions [13]. Different integrins bind distinct amino acid sequences on collagen, triggering various signaling pathways. For example, integrin α1β1 is essential for mesenchymal cells and inflammatory cells, including T lymphocytes [33]; integrin α2β1 modulates cell migration, proliferation, and survival [34]; integrin α10β1, expressed in cartilage, influences bone development [35]; and integrin α11β1, abundant in mesenchymal tissues during development, is essential for maintaining bone mass [36]. Tumor cells are highly proficient in secreting MMPs that cleave collagen, exposing RGD peptides that bind to αvβ3 integrins, enhancing invasion. RGD peptides, consisting of arginine, glycine, and aspartic acid, bind to integrin receptors, promoting cell adhesion [37]. The αvβ3 integrin is associated with tumor growth, neovascularization, and migration, with its expression levels correlating with cancer progression [38]. Due to their strong binding affinity for integrins, particularly αvβ3 integrins overexpressed in various cancer cells and vascular regions of the TME, RGD peptides have become pivotal tools in cancer therapy. Recent research has focused on utilizing RGD molecules to target αvβ3 and αvβ5 integrins, both of which play pivotal roles in tumor-induced neovascularization and angiogenesis, offering new potential avenues for cancer treatment [39].

The DDR family, a group of widely expressed tyrosine kinases, also recognizes collagen. Upon activation, DDR1 and DDR2 regulate cell proliferation, differentiation, and ECM responses, contributing to tumor progression through altered gene expression [40]. DDR1 acts as a collagen sensor, critical for cell migration, adhesion, and invasion, with its tumorigenic or antitumorigenic effects depending on factors such as tumor stage, type, downstream signaling, and the specific collagen isoform involved [41].

Additionally, MMPs cleave collagen at specific sites, releasing fragments that can be internalized through the mannose receptor (MR) family of endocytic receptors or *via* binding to β1 integrin [42]. The receptor-associated protein for urokinase plasminogen activator (uPARAP/Endo180) is a key collagen receptor involved in the internalization and degradation of collagen in mesenchymal cells and certain macrophages, and is a member of the MR family. uPARAP/Endo180 serves as a receptor for type V collagen and, to a lesser extent, other collagens (including types I and IV). Upon binding to uPARAP, collagen is internalized into clathrin-coated vesicles, enters early endosomes, dissociates from the receptor, and is directed to lysosomal compartments for degradation, while uPARAP is recycled back to the cell surface [43].

**Fig. 4:**
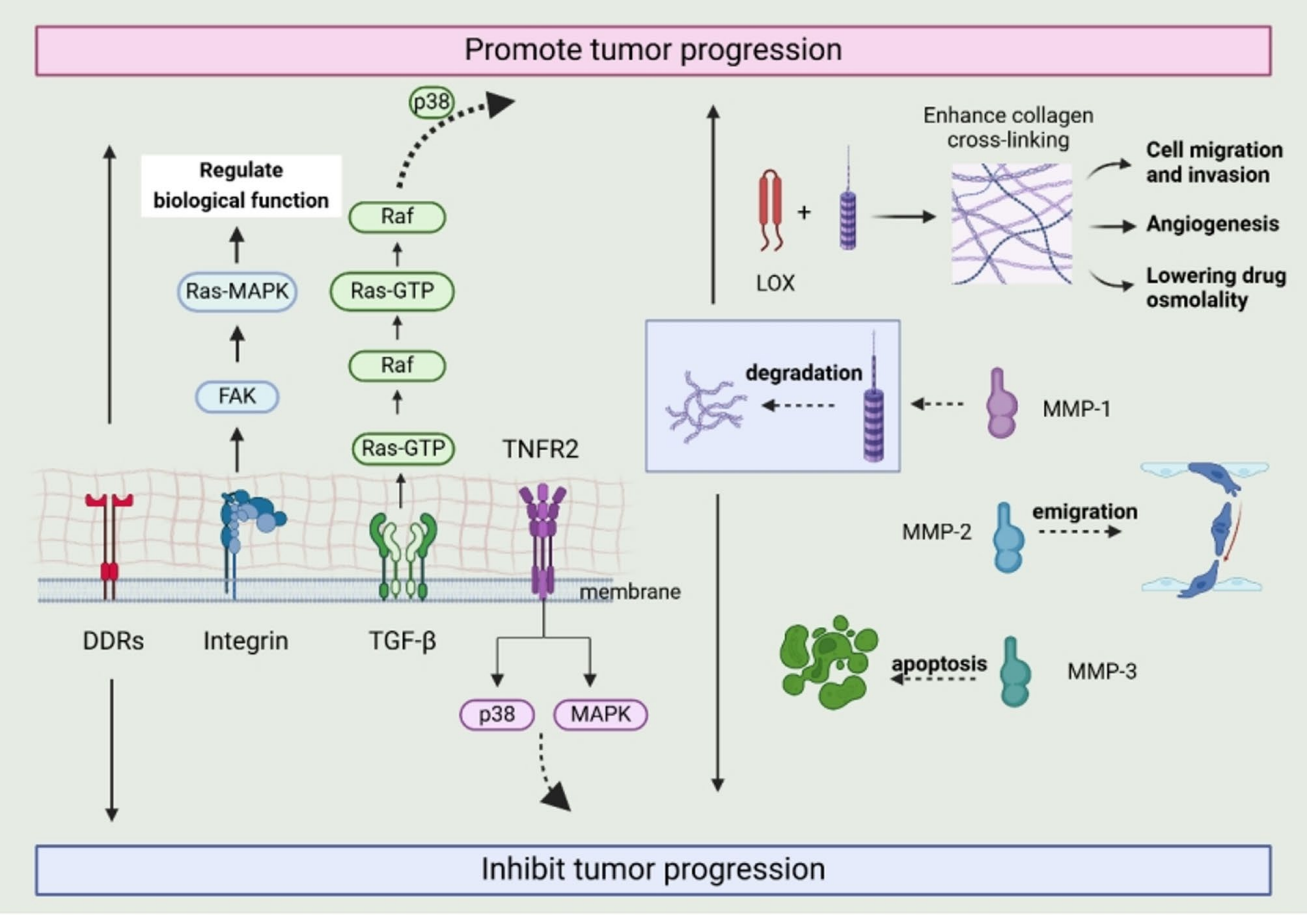
Effects of collagen binding to cell receptors on tumor cells. Upon integrin binding to collagen, FAK phosphorylation is triggered, initiating downstream pathways such as Ras-MAPK. The interaction between TGF-β and the Ras-Raf-MEK-ERK signaling axis enhances collagen synthesis, promoting cancer progression through p38 activation. Elevated collagen levels can suppress tumor growth and metastasis *via* the TNFR2/p38 MAPK pathway. The function of DDR1 may be either pro- or anti-tumor, depending on the tumor stage and type. Tumor cells secrete MMPs that degrade collagen: MMP-1 breaks down collagen fibrils, MMP-2 promotes cell migration, and MMP-3 is involved in apoptosis. The LOX family crosslinks collagen and elastin, modifying ECM stiffness, which in turn affects cell migration, invasion, angiogenesis, therapeutic resistance, and reduces drug permeability

Collagen–receptor binding activates various intracellular signaling cascades that significantly influence tumor cell behavior. For example, integrin–collagen attachment can initiate focal adhesion kinase (FAK) phosphorylation, stimulating Ras–MAPK pathways [44]. Collagen may also stabilize cytoplasmic β-catenin, facilitating its nuclear translocation and driving oncogenic transcriptional programs [45]. In melanoma, TGF-β interactions with the Ras–Raf–MEK–ERK cascades enhance collagen synthesis, further promoting cancer progression through p38 activation [46]. In fibrosarcoma, increased collagen suppresses tumor growth and metastasis by modulating TNFR2/p38 MAPK signaling. Integrin aggregation in tumor cells triggers FAK/Src phosphorylation, enhancing cell adhesion and migration through downstream intermediaries such as ERK2/MAPK, β-catenin, Rac, and Rho [47]. Specific β integrin subunits play key roles: β1 integrin accelerates the cell cycle and promotes therapy resistance, whereas β3 integrin associates with tumor cells exhibiting stem cell-like characteristics. For instance, COL IV can induce EMT by downregulating E-cadherin, upregulating N-cadherin, and increasing transcriptional repressors like Snail1, Snail2, and Sip1, thereby enhancing MMP-2 secretion and migratory capacity [48] (Fig. 4).

Tumor cells induce ECM remodeling, which involves dynamic changes in collagen, such as alterations in its quantity, stiffness, arrangement, cleavage state, and the processes of homo- and heterotrimerization. These restructured, collagen-rich matrices, in turn, influence tumor behavior, with key signaling mechanisms impacting tumor growth, metastasis, synthesis, secretion, assembly, crosslinking, degradation, and renewal [49]. MMPs secreted by cancer cells degrade collagen, modify its architecture, and release bioactive fragments or expose previously masked binding sites [50, 51]. Cleaved collagens (e.g., cCOL-I) and intact collagens (iCOL-I) exert opposing effects on protease activity, tumor growth, and metastasis. The ratio of cleaved to intact COL-I serves as a valuable prognostic marker, as increased ECM turnover promotes malignancy and metastatic spread [52].

Cancer cells can generate distinct homo-trimers of COL1 α1 chains (α1/α1/α1), which differ from the hetero-trimers (α1/α2/α1) produced by fibroblasts or normal cells. This shift results from the suppression of the COL1A2 gene due to promoter DNA hypermethylation. Although the exact signaling pathway remains unclear, Ras may play a role in downregulating α2 chain expression. Compared to hetero-trimers, COL1 homo-trimers in cancer cells strongly induce phosphorylation of DDR1, FAK, AKT, and ERK. This signaling remains active even after DDR1 inhibition, suggesting that other receptors may also be concurrently activated or a compensatory mechanism is at play. Additionally, the α3β1 integrin, associated with cancer cells, mediates this signaling transduction. These homo-trimers also influence the tumor immune microenvironment, promoting immune suppression that may exclude T cells. This effect is achieved by regulating the secretion of factors from cancer cells that recruit myeloid-derived suppressor cells (MDSCs) and is linked to a unique intratumoral microbiome [53].

Furthermore, collagen crosslinking in the TME can increase under the influence of enzymes such as LOX, which leads to a stiffer ECM that facilitates tumor invasion and metastasis [54]. LOX family members, including LOX and LOXL, crosslink collagen and elastin, altering tissue stiffness, cell migration, angiogenesis, and therapeutic resistance. They also elevate intratumoral fluid pressure, impairing drug penetration. In colorectal cancer (CRC), LOX enhances tissue stiffness and triggers the FAK/Src signaling cascade, accelerating tumor progression [55]. After exploring collagen’s interaction with tumor cells, how collagen influences immune cells and contributes to immune evasion in cancer is further examined.

### Collagen and immune cells

A diverse array of immune cells typically accumulates in collagen-rich regions of the TME. The three-dimensional ECM structure can act as a physical barrier to immune cell infiltration; for immune cells to penetrate deeper into tissues, they must actively navigate the ECM [56]. In healthy tissues, the collagen matrix is relatively porous, facilitating T lymphocyte and natural killer (NK) cell migration for immune surveillance. The diameter and density of collagen fibers influence immune cell migration, thereby determining the quantity and speed at which immune cells reach tumor sites.

**Fig. 5:**
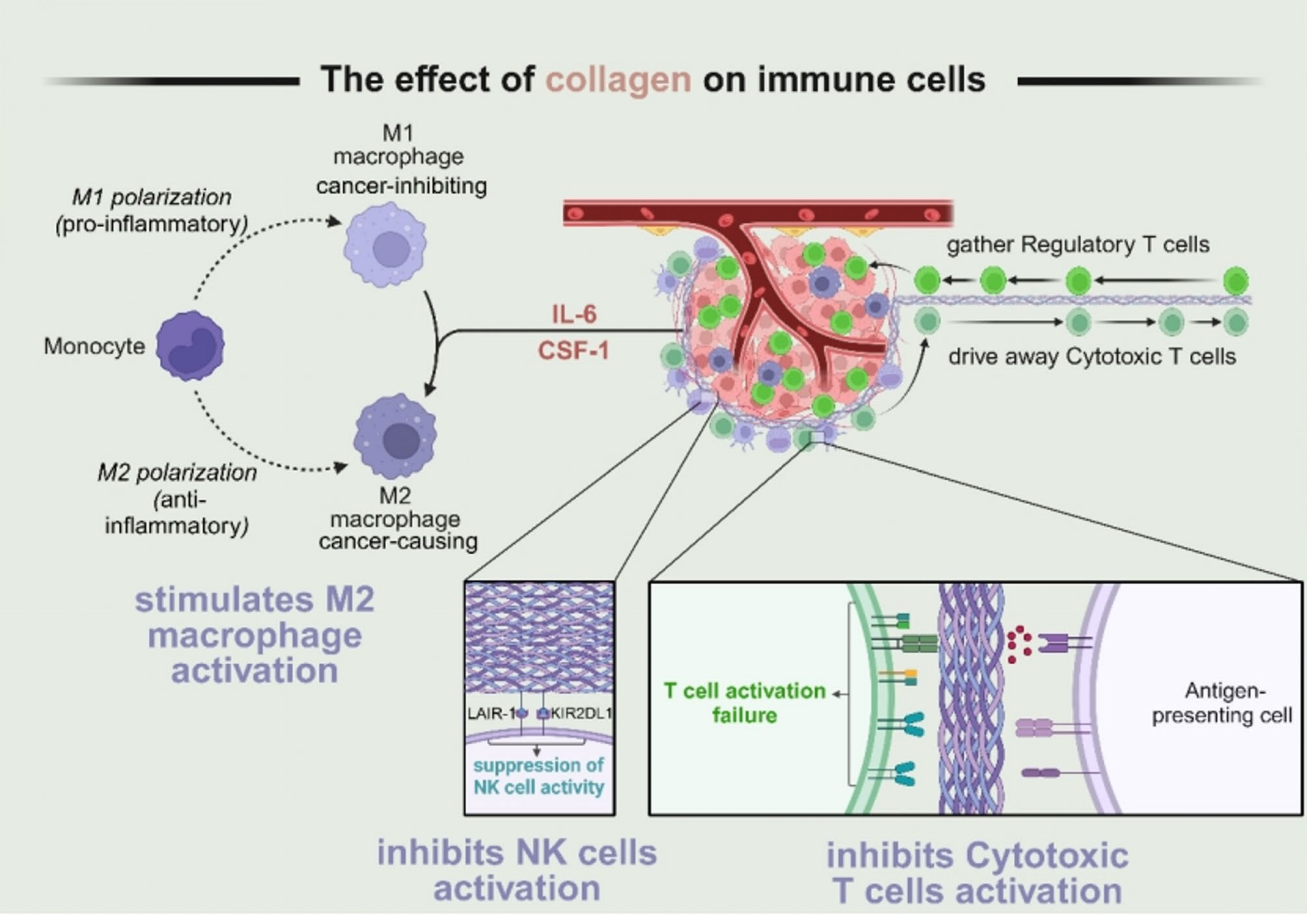
Effects of collagen on immune cells. Collagen regulates T-cell-mediated cancer cell elimination by influencing the migration of immune cells within the tumor, such as gathering regulatory T cells and dispersing cytotoxic T cells. The dense arrangement of collagen fibers reduces the proximity between antigen-presenting cells and T cells, hindering the formation of effective immunological synapses and decreasing T-cell activation. Collagen also regulates the balance between antitumor and protumor functions of macrophages through interleukin-6 (IL-6) and colony-stimulating factor 1 (CSF1). Increased macrophage recruitment and upregulated IL-6 expression can promote inflammatory responses, while inducing macrophage M2 polarization diminishes the capacity for an effective antitumor immune response. Additionally, collagen interacts with specific receptors on NK cells, inhibiting their activity. Created in BioRender.com

ECM components, including collagen, directly or indirectly shape T-cell migration, phenotype, and function (Fig. 5). High collagen density may hinder T-cell infiltration, guiding T-cells along collagen scaffolds in a proteolysis-independent manner. This orientation favors the accumulation of immunosuppressive regulatory T cells (Tregs) over cytotoxic T cells (CTLs). Tregs attenuate antitumor immunity, and their recruitment and activity can be modulated by collagen remodeling. Conversely, suppressing collagen crosslinking may alter tumor-associated macrophages (TAMs) and shift the balance between CD8^+^ T cells and Tregs, ultimately affecting T-cell activation. Additionally, collagen-producing macrophages consume environmental arginine and secrete proline and ornithine, creating metabolically unfavorable conditions for CD8^+^ T cells and further undermining cytotoxic responses [57].

T cells are lymphocytes characterized by distinct T-cell receptors and costimulatory molecules (e.g., CD4 or CD8). While CD8^+^ T cells exhibit potent cytotoxicity, CD4^+^ T cells coordinate broader immune functions, also conferring direct cytotoxic effects. High collagen density impedes T-cell motility; reduced pore size in dense collagen gels hinders T-cell migration [58, 59]. Dense collagen organization may also obstruct antigen-presenting cells from forming effective immunological synapses with T cells, thereby impairing T-cell activation [60]. Increased ECM stiffness suppresses T-cell proliferation and cytokine production associated with T-cell activity. In some tumor models, low rigidity and dispersed collagen fibers allow for improved T-cell infiltration, whereas high rigidity and thick, aligned collagen fibers limit T-cell trafficking and activation, indirectly reducing their antitumor capacity [61]. In tissue regeneration contexts, collagen scaffolds may promote an immunosuppressive microenvironment by increasing the CD4:CD8 ratio and steering CD4^+^ T cells toward a Th2 phenotype, which collectively diminishes T-cell cytotoxic potential [62].

Macrophages can polarize into classically activated (M1) or alternatively activated (M2) phenotypes, with the former typically exhibiting antitumor properties and the latter often promoting tumor progression [63]. Collagen plays a critical role in modulating the M1–M2 balance, influenced by factors such as IL-6 and CSF1. For example, the COL VI–ETP peptide can enhance macrophage infiltration and increase IL-6 expression, promoting inflammation. TAMs, primarily M2-like, can profoundly suppress antitumor immunity and are heavily influenced by ECM composition. Collagen density and mechanical properties affect macrophage phenotypes, with higher collagen density frequently driving a more immunosuppressive, pro-tumor macrophage profile [64].

Collagen also affects NK cells through multiple mechanisms. Collagen mineralization can enhance mucin-type O-glycosylation and sialylation on tumor cells, thickening the tumor glycocalyx and shielding it from NK-mediated cytotoxicity [65]. Binding of collagen to LAIR-1 on NK cells activates pathways involving SHP-1/2, reducing NK cell activity by limiting STAT1/4 phosphorylation and IFN-γ/TNF-α production [66]. Additionally, collagen may lower the expression of the natural cytotoxic receptor NKp30 and perforin, while increasing the inhibitory receptor KIR2DL1 on dendritic-like NK cells. These changes collectively reduce NK cell cytotoxicity [67]. Overproduction of collagen can create adhesion structures that protect tumor cells from NK-mediated killing. Interestingly, in collagen-induced arthritis models, adoptive transfer of NKp46^+^ NK cells exacerbates disease severity [68] although collagen may also mobilize and activate NK cells against pathological T helper cells [69]. Furthermore, blocking tumor collagen deposition significantly enhances NK cell cytotoxicity against MHC class I-deficient melanoma [66] suggesting that therapies targeting ECM–NK interactions could be effective in treating solid cancers.

Mast cells modulate collagen fibril formation by enhancing MMP-2 and MMP-9 activity, promoting CAF-mediated collagen lattice contractions [70]. Their interaction with CAFs contributes to fibrotic ECM accumulation [71] and mast cells directly participate in collagen fibril assembly, from procollagen and microfibers to the formation of fibrillar subunits [72]. Additionally, COL I can drive monocyte differentiation into dendritic cells (DCs), enhancing DC maturation and immune stimulatory functions [73]. While research on collagen’s impact on other immune cells remains limited, current findings highlight a promising area for further investigation.

Tumor cells employ various mechanisms to evade immune detection, including modifying their antigens and reshaping the TME to prevent immune cells from recognizing and eliminating malignant cells. In tumor tissues, COL I deposition parallels a fibrotic process that serves as a protective barrier against chemotherapy and immune effector cells, thereby strengthening immune evasion and drug resistance. Overexpression of COL I leads to fibrosis that supports tumor immune evasion and chemoresistance. Tumor cells may secrete collagen through EMT to shield themselves, or collagen may encapsulate tumor cells and compress local vasculature, limiting immune cell and therapeutic agent access.

Additionally, collagen–tumor interactions can stimulate tumor cell expression of immunosuppressive molecules (e.g., PD-L1), which binds PD-1 on T cells to inhibit their cytotoxic function [74, 75]. Collagen can also recruit immunosuppressive cells (e.g., Tregs) and shift the local cytokine milieu toward suppression, further inhibiting CTL or NK cell-mediated antitumor responses.

### Collagen and stromal cells

Stromal cells, particularly CAFs, are the primary source of collagen in the TME, actively secreting ECM proteins and soluble factors that inherently promote cancer progression. By producing LOX, CAFs crosslink collagen, restructure the stroma, and increase matrix stiffness, creating a favorable niche for tumor cell growth. Within the various CAF subpopulations, specific groups serve as the principal collagen producers. These CAF-derived ECM components are characterized by their high stiffness and density, primarily due to elevated collagen content. A high collagen density and increased fiber alignment have been linked to poor prognosis in numerous cancers, including breast, pancreatic, gastric, and oral squamous cell carcinoma [27].

COL I and COL III are the predominant collagens in connective tissue, forming stable hybrid fibers in varying ratios. Studies indicate that COL I and COL III exert distinct effects on CAF activation: stiffer COL I fibers apply stronger mechanical forces on cells, while integrin α1β1 shows a higher binding affinity for COL III than COL I [76]. Hybrid COL I–COL III fibers most effectively promote collagen synthesis. Hydrolyzed collagen and collagen peptides stimulate CAF proliferation, particularly low-molecular-weight peptides rich in proline and hydroxyproline, which penetrate deeper skin layers for more efficient tissue repair. At varying concentrations, collagen peptides protect human skin CAFs and keratinocytes by inducing anti-inflammatory responses against lipopolysaccharide insults [77]. Hydrolyzed collagen from defatted Asian sea bass (Asbs-HC) is non-cytotoxic to CAFs at concentrations from 25 to 1000 µg/mL, with the highest cell proliferation and migration observed at 1000 µg/mL. These findings suggest that Asbs-HC accelerates CAF proliferation and migration, while also exhibiting significant antioxidant activity, indicating potential applications in skin care and wound healing [78].

In addition to CAFs, VECs in the stroma have garnered increasing attention. One innovative study introduced a collagen–hyaluronic acid hydrogel platform with tunable mechanical plasticity [79]. Under high plasticity, this hydrogel triggers a cascade of effects in VECs, stabilizing integrin clusters and recruiting FAK. This leads to excessive cell contractility, downregulating vascular endothelial cadherin and destabilizing adherens junctions between VECs. Therefore, maintaining balanced matrix plasticity is critical for preserving cell–matrix adhesion and cell–cell contacts, which fosters vascular assembly and invasion. This research offers new theoretical insights into regenerative vascular medicine.

Distinct sources and forms of collagen have varying effects on VECs. Under hypoxic conditions, the collagen-modifying enzyme procollagen-lysine, 2-oxoglutarate 5-dioxygenase 2 in sarcoma cells modifies COL VI. Once this modified COL VI reaches the apical surface of pulmonary endothelial cells, it activates integrin β1 and colocalizes with Kindlin2, together stimulating F-actin interactions and polymerization. The resulting F-actin disrupts tight junctions, compromising the pulmonary endothelial barrier and facilitating sarcoma cells’ transendothelial migration [80]. In the cardiac microenvironment, endocardial endothelial cells (EECs) drive cardiac CAF proliferation and collagen production [81]. This process is largely mediated by factors such as endothelin-1, TGF-β, and angiotensin II, secreted by EECs, which regulate CAF proliferation, activation, and collagen synthesis through pathways involving protein kinase C (PKC) and mitogen-activated protein kinases (MAPK) [82].

Emerging microchannel collagen scaffolds show promise for guiding the organized arrangement of VECs. Experiments have demonstrated the formation of aligned tubular structures by VECs grown on these scaffolds, suggesting that the structure of collagen scaffolds significantly influences VEC morphology, arrangement, and function, with implications for angiogenesis. This provides a novel approach for constructing biomimetic vascular scaffolds [83]. Aligned nanofibrous collagen also regulates VEC morphology and migration, enhancing VEC performance in vascular grafts [84].

Different collagen family members exhibit unique functional characteristics. Collagen tripeptide can protect VECs by mitigating reactive oxygen species-induced transcriptional suppression, offering potential therapeutic benefits for atherosclerosis and other vascular dysfunctions [85]. In contrast, although collagen type VIII (COL VIII) is widely expressed in the vascular system and is involved in angiogenesis, vascular injury repair, arterial flexibility, and modulation of atherosclerotic plaque formation and ECM remodeling, it may inhibit VEC proliferation [86]. As a key basement membrane component, COL VIII serves as a ligand for integrin β1 and DDR1, influencing VEC migration and proliferation through specific signaling processes. Expression patterns observed in human diseases and animal models emphasize the critical role of COL VIII in vascular disorders, though its mechanistic details remain insufficiently understood. Further exploration of collagen’s role in non-tumor contexts may deepen our understanding of its function in VECs within the TME.

Collagen-related genes in tumor endothelial cells (TECs) exhibit distinct expression patterns that are integral to tumor angiogenesis. In patients with non-small cell lung cancer (NSCLC), the expression levels of PLOD1–3 and LOXL2—genes involved in collagen crosslinking—are significantly elevated in TECs compared to normal endothelial cells. Disrupting these genes impairs endothelial cell motility and proliferation, thereby affecting angiogenesis [87]. Gene enrichment analyses further suggest that collagen formation and ECM remodeling pathways are upregulated in TECs from prostate cancer (PCa), indicating that regulated collagen synthesis and structure in TECs influence their functional characteristics [88].

Collagen deposition alters matrix stiffness, shaping the microenvironment of VECs. In cases of heightened matrix rigidity, levels of angiogenesis-related factors in hepatocellular carcinoma (HCC) cells fluctuate, impacting VEC survival, proliferation, and migration. In HCC, Piezo1 mediates stiffness-induced angiogenesis, as evidenced by reduced tube formation and migration in human umbilical vein endothelial cells exposed to conditioned medium from Piezo1-knockdown HCC cells [89]. Additionally, local stiffness regulates calcium channels, influencing the transcription, translation, stability, and nuclear localization of hypoxia-inducible factor 1 (HIF-1)—a critical transcription factor for angiogenic genes. These findings highlight several pathways through which collagen-induced stiffness affects VECs. In a mouse orthotopic brain tumor model, inhibition of collagen crosslinking *via* LOX suppression reduces intratumoral collagen content, narrows tumor vessels, decreases vascular density, and lowers the expression of proangiogenic factors, collectively inhibiting tumor cell proliferation [90]. These interventions not only disrupt the tumor cell–endothelial cell relationship but also indirectly modulate endothelial cell function and influence angiogenesis. As the understanding of collagen in the TME progresses, the subsequent section focuses on its potential as a biomarker and explores its pivotal role in cancer.

### Collagen and cancer biomarkers

#### Definition and significance of biomarkers

A biomarker is an indicator that reflects the structure or function of a tissue, organ, or system, providing insight into normal or abnormal biological processes, conditions, or diseases [91, 92]. The standard definition describes a biomarker as “an objective measurement and evaluation of normal biological processes, pathogenic processes, or pharmacological responses to a therapeutic intervention” [93]. Biomarkers are typically classified as either endogenous or exogenous and further categorized based on their use for disease diagnosis, staging, or monitoring [94]. Ideal biomarkers are usually more cost-effective and easier to measure than final clinical outcomes, making the identification and discovery of valuable biomarkers a key objective for researchers.

The introduction of cancer biomarkers has revolutionized cancer diagnosis, marking a significant milestone in oncology. Various biomarkers have demonstrated clinical utility in early cancer detection and screening, such as alpha-fetoprotein (AFP), carcinoembryonic antigen (CEA), prostate-specific antigen (PSA), beta-human chorionic gonadotropin (β-Hcg), and carbohydrate antigen 19 − 9 (CA-19-9) [95]. However, their sensitivity and specificity often require improvement, as seen with CA125 in ovarian cancer screening, PSA testing in PCa, and CEA measurement in gastric cancer (GC) [96, 97, 98]. These markers do not always reliably differentiate benign from malignant tumors and are only useful in specific contexts for certain tumor types [99]. Therefore, the discovery of a biomarker with high sensitivity and specificity remains a crucial challenge in cancer diagnosis.

### Progress of collagen as a cancer biomarker

ECM remodeling serves as a key indicator for predicting cancer development. Collagen deposition, a key component of the ECM, often leads to fibrosis—a notable sign of tumor progression with substantial clinical significance [100]. For example, continuous accumulation of types I and III collagen in the liver causes hepatic fibrosis, which eventually predisposes individuals to HCC [101, 102]. Severe fibrosis has been positively correlated with the onset of HCC [103, 104]. Recent studies have shown significant changes in tumor and stromal cell activity during glioblastoma (GBM) progression, resulting in increased deposition of ECM components such as collagen, fibronectin, and tenascin-C. Simultaneously, hyaluronic acid (HA) becomes more abundant, with a shift toward lower molecular weight forms. Collagen VI plays a pivotal role in this process. In bevacizumab-resistant cultured models, collagen VI secretion is markedly enhanced, which strengthens the previously soft HA matrix by binding to specific integrin subtypes, triggering mechanotransduction signaling. This process activates the β-catenin pathway, promoting the expression of the mesenchymal marker ZEB1 and accelerating tumor cell invasion. Inhibiting collagen VI function has been shown to significantly reduce hypoxia-driven invasion and the expression of mesenchymal markers [105]. Collagen VI is a prominent biomarker in U87MG tumors and is found in the pathological blood vessels of human gliomas. GBM cells secrete collagen VI to enhance their invasive capabilities, with the interaction between SNAP25 and collagen VI playing a vital role in the adhesion of GBM cells expressing SNAP25 on their surface [106]. Similarly, collagen deposition in the oral mucosa, leading to oral submucosal fibrosis, poses a significant risk for oral cancer [107]while excessive collagen accumulation in the breast is considered a key driver of breast cancer [108]. In cervical cancer, studies analyzing the spatial distribution of collagen have identified parameters such as collagen density and disrupted collagen crosslinking as key factors for quantitatively categorizing precancerous stages [100]. Thus, alterations in collagen can serve as reliable indicators of malignancy.

Ongoing research into tumor-associated collagen has identified several collagen family members as potential biomarkers for cancer detection and diagnosis [2]. For instance, Moa Lindgren et al. discovered that COL IV outperforms the widely used biomarker “circulating CA15-3 (cCA15-3)” in both specificity and sensitivity for detecting metastatic breast cancer (mBC) [109]. In addition to mBC, Niloufar Salimian et al. found that changes in the gene expression of collagen family members, particularly COL1A1 and COL11A1, could serve as biomarkers for CRC and GC [110]. In HCC, AM Attallah and colleagues combined collagen III with MMP1 to develop an HCC-ABC detection method, proving its potential as an early biomarker for HCC [111]. These findings have opened avenues for further exploration in other cancers, including pancreatic ductal adenocarcinoma (PDAC), renal cell carcinoma (RCC), small cell lung cancer (SCLC), and PCa [100, 112]. While collagen family molecules show significant promise in cancer detection, further investigation into their precise mechanisms is necessary to facilitate their widespread clinical application [19].

Emerging evidence highlights the pivotal role collagen plays in predicting clinical prognosis, particularly in the context of therapeutic responses. Factors such as collagen type, structure, distribution, and the degree of fibrosis are closely linked to treatment outcomes, especially in immunotherapy. Collagen family molecules, by stiffening the ECM and creating a physical barrier, can hinder the penetration of immune cells, chemotherapeutic agents, CAR-T cells, and even gamma irradiation into the tumor interior, reducing treatment efficacy. Additionally, collagen can suppress hydroxyl radical (*OH)-induced apoptosis, creating a more favorable environment for tumor cell survival [11, 113, 114]. Higher collagen density, tighter collagen arrangement, elevated expression of collagen-related genes, and reduced immune cell infiltration are often associated with poor immunotherapy responses [41, 64, 115–117]. This suggests that collagen may serve as a predictive biomarker for cancer immunotherapy outcomes.

Current research further supports the potential of collagen (particularly COL I) as a prognostic biomarker in several cancers, including GC, breast cancer, PDAC, SCLC, bladder cancer, PCa, CRC, and HCC [19, 100, 118]. For instance, Kaplan–Meier analyses by Yihuan Chen et al. have shown a strong correlation between elevated collagen expression and poor prognosis in GC, indicating that collagen family molecules may reliably predict clinical outcomes [119]. Similarly, in breast cancer, type I collagen family molecules have been validated as effective prognostic biomarkers by Wenjie Shi and colleagues [108]. Given the abundance of collagen in PDAC tissues, the correlation between collagen levels and prognosis in PDAC is particularly strong, as supported by Jeppe Thorlacius-Ussing’s study [120]. Further supporting this, Jie Mei and coauthors proposed a tumor classification framework that categorizes tumors into three subtypes based on collagen activity and immune infiltration: “soft&hot” (low collagen activity, high immune infiltration), “armored&cold” (high collagen activity, low immune infiltration), and “quiescent” (low collagen activity, low immune infiltration) [121]. This classification has proven effective in predicting prognosis across 13 cancer types, including NSCLC, ovarian serous cystadenocarcinoma (OV), and cervical cancer, while also accurately forecasting immunotherapy responses for most cancer types [121]. Ongoing investigations are also exploring the potential of collagen as a prognostic biomarker for gliomas and head and neck cancers [122]. Collectively, these findings suggest that collagen could be a reliable prognostic biomarker across a variety of malignancies, aiding clinical decision-making. Future research into the interplay between collagen and immunotherapy responses may lead to more precise predictive tools for personalized immunotherapy, ultimately refining cancer treatment strategies.

### Collagen meets cancer immunotherapy

#### Combined collagen inhibition and immune checkpoint blockade

As immune checkpoint inhibitors (ICIs) have become more widely utilized in recent years, a subset of patients has exhibited either primary or acquired resistance, rendering single-agent checkpoint blockade insufficient to restore a functional immune cycle. These patients often derive limited benefit from ICI monotherapy [123]. Thus, combining ICIs with other synergistic therapies to enhance antitumor effects has become a prominent research focus. Indeed, combinations of ICIs with chemotherapy, radiotherapy, interferon, CAR-T therapy, and co-stimulatory agonists have shown enhanced antitumor efficacy and improved response rates [124]. For example, combining nivolumab with everolimus has proven effective in patients with advanced RCC who were previously treated with sunitinib or sorafenib; co-administering atezolizumab with paclitaxel has extended survival in patients with advanced triple-negative breast cancer; and adding pembrolizumab to chemotherapy has improved outcomes in metastatic NSCLC [125–127].

As understanding of the TME evolves, collagen has garnered increasing attention in cancer immunotherapy. Multiple targets involved in collagen synthesis modulation—such as MMP, LAIR-1, DDR1, HSP47, and LARP6—have been identified [29, 128, 129] sparking interest in collagen’s untapped potential in cancer immunotherapy. Preliminary findings suggest that combining collagen-targeted strategies with ICIs could open new treatment avenues.

Elevated collagen levels are closely associated with CD8^+^ T-cell exhaustion in lung cancer, marked by increased numbers of exhausted CD8^+^ T cells and resistance to PD-1/PD-L1 inhibition. This occurs when collagen interacts with LAIR-1, which suppresses T-cell activity *via* SHP-1 signaling [24]. Additionally, the N-terminal propeptide of type III collagen (PRO-C3) can indicate the release of collagen precursors during collagen formation, while C3M quantifies MMP-degraded collagen. In vitro data demonstrate that TGF-β-induced CAFs release PRO-C3, suggesting its potential as a marker for assessing CAF activity and collagen-rich peritumoral stroma, which are linked to ICI resistance [22].

Tumors often create an immunosuppressive TME to evade immune surveillance, characterized by reduced tumor antigen recognition, the accumulation of immunosuppressive cells (e.g., Tregs, B regulatory cells, MDSCs, and M2-polarized TAMs) [130]and the upregulation of co-inhibitory checkpoints. Dysregulated ECM can also impair ICI efficacy by forming a dense stromal barrier that blocks ICI penetration or altering ECM stiffness or degradation patterns, leading to immune cell “traps.” Furthermore, hypoxic conditions can shift angiogenesis, further hampering ICI activity. Increased collagen deposition in solid tumors (e.g., lung tumors) can contribute to PD-1/PD-L1 resistance [24]. Strategies aimed at reducing collagen levels could enhance T-cell infiltration into tumors, potentially reversing ICI resistance.

Recently, targeting the LAIR-1–collagen axis has emerged as a novel checkpoint blockade strategy [131]. Combining anti-PD-1 antibodies with LAIR-1 inhibitors significantly improves treatment outcomes, as LAIR-1 blockade enhances CD4^+^ and CD8^+^ T-cell infiltration, potentiating anti-PD-L1 therapy in humanized xenograft models of colon and pancreatic cancer [131, 132]. In mouse pancreatic tumor models, bacteria-based delivery of collagenase reduces collagen density and enhances ICI efficacy [133]. Additionally, DDR2 signaling may mediate collagen’s impact on immunotherapy response, as joint administration of anti-PD-1 antibodies and a DDR2 inhibitor boosts CD8^+^ T-cell infiltration and decreases tumor burden in multiple mouse cancer models [134]. Similarly, inhibiting TGF-β can reduce intratumoral collagen levels and improve checkpoint inhibitor outcomes [135]. Notably, therapies combining ICIs with IL-2 linked to the vWF-A3 domain (a collagen-binding motif) have shown promising safety and efficacy in breast cancer models [136].

Cetuximab, an anti-EGFR antibody, retains better in collagen-rich tumors, enhancing its therapeutic effects [137]. Moreover, elevated serum levels of PRO-C3 have been associated with poor prognosis in patients with metastatic melanoma treated with either anti-CTLA-4 [138] or anti-PD-1 [139] therapies, while high collagen levels in lung cancer correlate with suboptimal responses to anti-PD-1/PD-L1 treatment [24]. The predictive value of collagen extends beyond tissue, as serum collagen fragments offer a noninvasive method for patient stratification to optimize ICI efficacy [139].

The transforming growth factor-β (TGF-β) signaling pathway is a central regulator of CAF activity and collagen production [140]. Therapeutically targeting TGF-β effectively reduces collagen deposition and inhibits tumor progression. Combination regimens involving chemotherapy, radiotherapy, or molecularly targeted drugs with TGF-β inhibition have shown promising potential to enhance antitumor efficacy by remodeling the TME [141]. In TNBC, BiTP—a bispecific antibody targeting both the TGF-β pathway and human PD-L1—significantly reduces stromal collagen accumulation, enhances T-cell infiltration, diminishes immunosuppressive components, and exhibits potent antitumor activity [142]. In CRC, simultaneous administration of a TGF-β blocker and an anti-PD-L1 antibody promotes CD8^+^ T-cell infiltration and elicits a strong antitumor response [143]. Vactosertib, a TGF-β–pathway inhibitor, combined with nal-IRI plus 5-fluorouracil/leucovorin, has been shown to extend overall survival in PDAC [144–146]. Over the past three years, numerous TGF-β signaling-targeting interventions—either alone or in combination—have entered clinical trials, including Fresolimumab (GC1008), Galunisertib (LY2157299), Trabedersen (AP12009), and Vactosertib [141]. Continued exploration of TGF-β’s multifaceted roles and the development of TGF-β-based combination therapies could offer renewed hope for cancer individuals.

Losartan, an angiotensin II receptor antagonist clinically approved for inhibiting type I collagen production by CAFs, has been shown to reduce hepatic and peri-tumoral fibrosis. It also significantly enhances tumor regression induced by anti-PD-1 therapy. Although losartan does not directly increase T-cell activity, it substantially improves the infiltration of effector CD8^+^ T cells into HCC compared to PD-1 blockade alone [147]. Additionally, losartan has demonstrated a significant enhancement of the anti-tumor efficacy of intratumoral injections of oncolytic herpes simplex virus and liposomal doxorubicin, and it may improve the efficacy of nanotherapies in patients with desmoplastic tumors [148]. When combined with pH-sensitive, disintegrable liposomes containing paclitaxel (PTX-Cl-Lip), losartan promotes paclitaxel accumulation and enhances its anti-tumor efficacy in the 4T1 mouse model [149].

Talabostat, another agent that inhibits type I collagen accumulation, has been proven effective and safe in a clinical trial in combination with pembrolizumab (an anti-PD-1 antibody) for treating advanced solid tumors. The combination of anti-type I collagen agents with ICIs represents a promising and more effective strategy for combating tumors [150].

### Safety evaluation of collagen inhibition plus immune checkpoint blockade

Most collagen-based anticancer therapies remain in preclinical development, limiting their clinical impact. Efforts to target collagen often yield contradictory results regarding drug delivery and therapeutic efficacy, especially when collagenase-based approaches lead to significant adverse reactions or paradoxical outcomes. Existing clinical trials are limited and primarily focus on specific pathways or receptors, with indirect collagen-targeting strategies—often involving multiple mechanisms—remaining under debate [13].

Data remain insufficient to rule out the possibility of unique immune-related adverse events (irAEs) or severe life-threatening side effects when combining collagen-targeted therapies with ICIs. Additionally, the potential negative effects of targeting collagen must be carefully considered. One study indicates that myofibroblastic CAFs (myCAFs), a subtype of CAFs enriched in liver metastases, exert mechanical restrictions on tumors by secreting type I collagen, which inhibits tumor dissemination [151]. Moreover, in bone, the primary sources of COL I production are Fap^+^ and Fsp1^+^ cells. Specific deletion of Col1 in Fap^+^ cells leads to lethality during late embryonic stages, accompanied by skeletal developmental defects, hemorrhage, and edema. In adult mice with specific deletion of COL1 in Fsp1^+^ cells, osteogenesis imperfecta-like phenotypes occur, characterized by multiple spontaneous fractures and bone fragility. This study suggests that embryonic skeletal development primarily relies on Fap^+^ cells for Col1 synthesis, while Fsp1^+^ cells are essential for maintaining and repairing the adult skeleton [152]. Another important study found that the COL III pro-peptide can effectively inhibit CAF activation, significantly suppressing the growth of breast cancer tumors [153]. Additionally, Di Martino demonstrated that applying polymerized COL III combined with sponge materials to tumor resection areas significantly inhibited the recurrence and proliferation of residual cancer cells after surgery. These findings suggest that COL III may inhibit tumor progression by blocking pro-cancer stromal signaling pathways or by physically isolating residual cancer cells. Furthermore, during PDAC progression, COL1 deficiency in myofibroblasts correlates with an increased population of MDSCs. These MDSCs express high levels of CD206, F4/80, arginase-1, CCL2, and interleukin-18, potentially contributing to the establishment of an immunosuppressive microenvironment in PDAC. These MDSCs can also inhibit T and B lymphocyte functions *via* arginase-1 and CD206, thereby promoting PDAC metastasis [154]. The role of collagen in tumor suppression is a critical factor that must not be overlooked when developing therapeutic strategies. Rigorous clinical validation is essential to confirm the safety of co-targeting collagen and immune checkpoints.

### Novel directions for collagen-targeted therapy

Targeting the mechanical interface between the ECM and hepatic cancer cells has introduced new paradigms in cancer treatment, particularly for HCC. For example, targeting the matricellular protein agrin has been shown to reduce mechanical signaling and tumor angiogenesis, thereby inhibiting oncogenic signaling and suppressing tumorigenesis [155]. Agrin is secreted by hepatic stellate cells following PDGF stimulation [156]. Sorafenib, by inhibiting PDGF receptors, reduces inflammation, fibrosis, HCC development, and agrin secretion. This has positioned agrin as a promising novel target for HCC, leading to the question: could collagen be similarly utilized?

Current collagen-targeted therapies primarily rely on two approaches [136]: linking a collagen-binding domain (CBD) or collagen-binding protein (CBP) to the drug at an active chemical site to form collagen-binding conjugates, or designing therapeutic fusion proteins that incorporate CBD/CBP. Several immunotherapies, such as therapeutic antibodies targeting EGFR [137] PD-L1, or CTLA-4, and cytokines like IL-2 and IL-12, can be modified with CBD or CBP [153, 157]. Enhancing CBD markedly increases collagen affinity, facilitating faster drug accumulation within tumors, prolonged retention, and controlled release [137]. This modification also helps mitigate toxicity, emphasizing CBD’s potential in reducing side effects caused by therapeutic antibodies.

A recent report highlighted a CBD-SIRPαFc conjugate as a novel tumor-targeting CD47 inhibitor [158]. By using a short Sulfo-SMCC linker, the collagen-binding motif TKKTLRT was integrated with the SIRPαFc fusion protein, allowing the blockade of the immune checkpoint molecule CD47 while boosting phagocytosis-mediated tumor inhibition. Due to its strong collagen affinity, CBD-SIRPαFc accumulates more rapidly and persists longer at tumor sites, potentially preventing off-target CD47 blockade and its associated adverse reactions. In a nude mouse A549 xenograft model, CBD-SIRPαFc exhibited superior antitumor efficacy compared to unmodified SIRPαFc, with increased macrophage infiltration and activation. Various antibodies and cytokines similarly modified by CBD or CBP have shown improved therapeutic indices across multiple tumor models, underscoring the potential of CBD/CBP-based collagen-targeting to enhance immunocytokine therapy efficacy while mitigating its common adverse effects [159].

The tumor-targeting capability of collagen-linked cancer immunotherapeutics is strongly supported by evidence showing higher intratumoral drug concentrations, longer drug retention, and improved antitumor efficacy [160]. Healthy tissues benefit as well, with reduced drug uptake and fewer peripheral side effects. The CBD platform can be flexibly incorporated into different immunotherapies, making collagen targeting adaptable across numerous tumors and compatible with various immunotherapeutic agents. One promising example involves targeting TME collagen in combination with ICIs, such as the MRC2 collagen receptor, which is highly expressed on immunosuppressive ECM myCAF cells. Its deletion enables enhanced CD8^+^ T-cell infiltration and increases ICIs sensitivity in a murine breast cancer model [161]. Thus, collagen-targeting represents a novel strategy to improve immunotherapies, including checkpoint inhibitors [61]TIL-based approaches [162]and cancer vaccines [163]. Future collagen–cancer immunotherapy conjugates hold significant promise for advancing anticancer therapies.

Collagen significantly impacts the efficacy of nanoparticle-based therapies in animal models [164]. For example, combining losartan nanoparticles with other chemotherapeutics or treatment regimens can substantially reduce collagen content, thereby enhancing tumor penetration and improving therapeutic outcomes [165]. Collagen also serves as a drug carrier or target site: hybrid collagen–cell-penetrating peptide carriers enhance enzyme resistance [166] and combining cetuximab single-chain fragments with CBD has shown promising anticancer results [167]. These findings highlight the potential of collagen-targeted therapies. Proline metabolism, central to collagen translation, supports cancer cell growth and shapes a tumor-promoting TME. Inhibiting proline metabolism to block collagen translation represents a promising therapeutic approach. However, removing collagen can sometimes relieve tumor cells from ECM constraints, accelerating disease progression [168]. This emphasizes the need for careful balance in ECM reduction or disruption to optimize antitumor effects and improve treatment responses.

Further insights may come from cancer-specific fibroblasts, which coexpress COL11A1 and CAF markers that are unique to tumors but absent in healthy tissue [169]. These fibroblasts play a pivotal role in ECM remodeling, where high levels of COL11A1 activate TGF-β signaling, leading to CAF activation, tumor progression, and poor clinical outcomes [169]. Given COL11A1’s pro-tumor role, it could become a novel target in cancer immunotherapy.

Additionally, factors such as patient age and estrogen levels must be considered when evaluating targeted collagen therapy. Age can directly impact immune system function and overall treatment response. Research indicates that older patients typically face higher risks of complications, which can influence the tolerance and efficacy of targeted collagen therapies [170]. Moreover, certain cancers may exhibit different biological behaviors across age groups, affecting the adaptability of collagen-targeted therapies [171].

Estrogen plays a significant role in cancers such as breast and uterine cancer, influencing tumor growth and metastasis. Studies suggest that estrogen may alter the TME by affecting collagen metabolism and remodeling, thereby influencing treatment success [172]. Thus, fluctuations in estrogen levels may impact the response to targeted collagen protein therapy [173].

### Clinical trials targeting collagen

Although collagen-targeted approaches for cancer therapy show promise, clinical practice highlights that these new treatments still face challenges related to safety and efficacy. To address this, clinical trials focused on evaluating the feasibility of these methods are summarized, aiming to bridge existing therapeutic gaps and offer renewed hope to patients. The focus is on drugs that target collagen-related processes at three critical stages: regulating CAFs, intervening in collagen synthesis, and modulating collagen degradation.

CAFs become abnormally activated during tumorigenesis, secreting substantial amounts of collagen and other factors that facilitate tumor expansion. Thus, anticancer therapies targeting CAFs have attracted considerable interest. Palbociclib, a CDK4/6 inhibitor, disrupts the key processes by which CAFs promote tumor cell proliferation through kinase inhibition. In a Phase II clinical study involving postmenopausal Japanese patients with ER-positive, HER2-negative advanced breast cancer, Palbociclib combined with Letrozole demonstrated both efficacy and tolerability, providing long-term supportive evidence for its use in this population. Additionally, patients with advanced gastrointestinal stromal tumors resistant to imatinib and sunitinib showed varying degrees of response to Palbociclib, suggesting its antitumor potential [174, 175]. Talabostat (Val-boroPro), the first clinical inhibitor of protease activity in tumor-associated CAFs, was tested in a Phase II trial for metastatic CRC. This study provided the first proof that physiologically inhibiting FAP activity in CRC is feasible [176]. Talabostat, in combination with docetaxel, also demonstrated antitumor efficacy and manageable safety in advanced NSCLC models, establishing a foundation for future stroma-targeted research [177]. Finally, Losartan, widely known for its antifibrotic properties, inhibits CAF proliferation and collagen deposition in the lungs. A 12-month prospective, non-controlled pilot study identified Losartan as a promising therapeutic agent for idiopathic pulmonary fibrosis, highlighting its low-toxicity profile [178].

Regarding collagen synthesis, several drugs targeting this fundamental process are under clinical investigation. Pirfenidone, an orally administered anti-inflammatory and antifibrotic agent, is effective against idiopathic pulmonary fibrosis and has shown antineoplastic potential in neurofibromatosis type 1-associated plexiform neurofibromas by inhibiting CAF proliferation and collagen production [179]. A Phase II trial confirmed Pirfenidone’s therapeutic effects and safety in these diseases [180]. While Pirfenidone demonstrated promise in slowing tumor progression and preserving quality of life, adverse effects emerged as key areas for improvement in future clinical studies. These findings emphasize the need for more potent treatments and refined clinical strategies.

This review further focuses on the “final destination” of collagen—its degradation. In cancer progression, the breakdown of collagen often becomes imbalanced, either through overdegradation, which facilitates metastasis, or insufficient degradation, which increases TME pressure and impedes immune cell function. As a result, agents that regulate collagen degradation have garnered significant attention. Marimastat, a broad-spectrum MMP inhibitor, directly targets the MMP active site, helping restore normal collagen degradation and preventing tumor cells from exploiting a destabilized stroma. Halofuginone, another inhibitor that specifically blocks COL I and MMP-2 gene expression, disrupts neovascularization and prevents intimal hyperplasia at vascular anastomoses. A clinical trial evaluating topical Halofuginone in AIDS-related Kaposi’s sarcoma demonstrated significant tumor regression in select patients, with tumor size reduction and improved appearance, suggesting notable antitumor activity and acceptable safety and tolerability [181]. However, the study also identified various efficacy and safety constraints, prompting further efforts to optimize topical Halofuginone regimens, explore combination treatments, and investigate other localized therapeutic options.

Currently, few clinical trials target collagen, and available data suggest that many agents have limitations in therapeutic outcomes and safety. Larger-scale, more comprehensive research is needed to clarify their potential benefits and risks. Furthermore, detailed clinical trials are essential to fully explore how collagen-based strategies could be integrated into cancer immunotherapy, considering collagen’s distinctive role within the oncological landscape.

### Future directions and challenges

As a major ECM component, collagen plays a pivotal role in all stages of tumorigenesis, from cellular proliferation to invasion and metastasis. Collagen remodeling frequently occurs during cancer initiation or progression, offering the potential for detection through imaging and pathological evaluation, thus presenting promising opportunities for clinical diagnosis and staging. Furthermore, biochemical collagen metrics have emerged as powerful prognostic biomarkers. As previously mentioned, some researchers have developed a collagen-based tumor classification system, with substantial clinical evidence showing that inhibiting collagen can enhance the efficacy of immunotherapy.

In summary, collagen holds significant promise for advancing cancer diagnostics and therapeutics, potentially driving key breakthroughs in tumor immunology and possibly leading to novel strategies for cancer treatment. However, several challenges must be addressed before collagen-based therapies can be routinely applied in clinical practice. Currently, only a few collagen-targeted drugs are approved for clinical use, with most remaining in preclinical or investigational stages. The complex relationship between collagen and cancer is not yet fully understood. While existing data indicate that collagen may act as a promising biomarker and an enhancer of immunotherapy, the underlying mechanisms—spanning multiple signaling pathways and cell types—remain unclear, hindering broader clinical adoption in diagnostics and therapy. Additionally, most collagen-focused targets are still in the early stages, lacking sufficient foundational and clinical evidence to confirm the safety and efficacy of combining collagen-targeted interventions with immunotherapies. Tumor heterogeneity presents another challenge, as collagen expression can vary significantly across different cancer types, meaning that no single collagen-based target is universally effective.

Therefore, defining the role of collagen and its interactions with cancer, exploring its crosstalk with immunotherapies, and conducting large-scale randomized clinical trials and foundational research are crucial to confirming collagen’s safety and efficacy in enhancing immunotherapy. At the same time, the potential negative effects of targeting collagen must be carefully considered in tumor treatment. An important issue that requires immediate attention is how to accurately stratify patients to maximize the benefits of collagen targeting in combination with ICIs while minimizing adverse effects. Moreover, while most current research emphasizes collagen interactions with T cells, B cells, macrophages, and NK cells in the EMT, limited studies have explored the relationship between collagen and DCs. Future investigations into DC–collagen crosstalk may offer valuable insights into collagen’s multifaceted immunomodulatory mechanisms.

## Conclusion

In conclusion, collagen’s pivotal role in tumor immunology has become increasingly apparent. It not only serves as the structural foundation of the ECM but also emerges as a promising therapeutic target for cancer immunotherapy, an active participant in cancer initiation and progression, and a potential biomarker for clinical use. Collagen represents a key milestone in tumor immunology, acting as a crucial pivot that could unlock more effective cancer immunotherapy strategies and enhance biomarker-based predictions.

Nevertheless, two critical unknowns remain: the intricate relationship between collagen and tumors and the full scope of collagen’s mechanism of action during cancer immunotherapy. Unanswered issues include potential side effects of integrative collagen–immunotherapy strategies, how patient heterogeneity may alter responsiveness to collagen modulation, whether DCs interact with collagen during EMT, and the role of collagen in cancer vaccines or adoptive cell therapies. As the mystery of collagen steadily unravels, further evidence should confirm its function as a potent cancer biomarker and reveal new therapeutic targets, thereby offering innovative avenues for cancer detection, diagnosis, and treatment.

**Table 1:** Classification table for cancer immunotherapy

Type of immunotherapy	Representative drugs/examples	Principle
Cytokine Therapy	IFN-α, IL-2	By employing cytokines (such as IL-2, IFN-α, and GM-CSF) to activate the immune system, thereby enhancing the functionality of immune cells and promoting antitumor responses.
Monoclonal Antibodies	Trastuzumab, Keytruda	By directly acting on tumor cells or immune cells to inhibit tumor growth or strengthen the immune response.
Immune Checkpoint Inhibitors	Targeting PD-1: Pembrolizumab Targeting PD-L1: Atezolizumab Targeting CTLA-4: Ipilimumab	By inhibiting immune checkpoints (for example, PD-1, PD-L1, and CTLA-4) to relieve immunosuppression and thereby improve the clearance of tumor cells by immune cells.
Adoptive Cell Transfer	Kymriah, Yescarta	By genetically modifying a patient’s immune cells (e.g., T cells or B cells) so that they can recognize specific tumor antigens and generate a targeted antitumor immune response.
Oncolytic virus therapies	talimogene laherparepvec	By using genetically engineered viruses to infect tumor cells, simultaneously activating the host immune system and directly killing cancer cells, thus creating a proinflammatory environment that augments antitumor immunity.
Cancer Vaccines	Sipuleucel-T, HPV	By stimulating the body’s immune system, especially T cells, which in turn produce a highly specific antitumor response.

**Table 2:** Schematic table of collagens that May serve as biomarkers

Cancer Type	Collagen Type	Possible biomarkers
Breast Cancer	Type I Type III Type IV Type V Type VI Type VIII Type XI Type XIV	1.degradation products of type I collagen 2.MMP-generated fragments of type I collagen in serum MMP-generated type III collagen fragment the type IV collagen the type V collagen MMP-generated type III collagen fragment the type collagen type VIII 1.the expression level of Collagen Type XI 2.the expression of procollagen XI Alpha 1 Chain the expression of type XIV collagen
Lung Cancer	Type I Type III Type V Type VI Type VII	1.the expression level of collagen I 2.alkaline phosphatase of collagen I in serum 3.MMP-generated fragments of type I collagen in serum 4.degradation products of type I collagen procollagen type III N-peptide in serum the expression level of collagen type V MMP-generated type III collagen fragment the expression level of type VII collagen
Pancreatic Cancer	Type I Type III Type IV Type VI Type XI Type XVIII	degradation products of type I collagen the propeptide of type III collagen in serum 7 S domain of type IV collagen in serum 1.MMP-generated type III collagen fragment 2.serum collagen type VI alpha 3 3.the expression level of collagen type XI the expression level of Collagen Type XI Alpha 1 Chain the expression of type XVIII collagen
Hepatocellular Carcinoma	Type I Type III Type IV	the expression level of collagen type I alpha 1 type III collagen in serum 1.7 S domain of type IV collagen in serum 2.serum N-terminal pro-peptide of type IV collagen 7 S domain
Gastric Cancer	Type I Type V Type XV	the expression level of collagen type I alpha 2 the expression level of collagen type V alpha fragments of collagen XV collagen alpha1 in urine
Colorectal Cancer	Type I Type IV Type V Type VIII Type XI Type XVIII	degradation products of type I collagen the differential localization of the type IV collagen alpha5/alpha6 chains the expression level of collagen type V alpha 2 collagen type VIII alpha 1 chain the expression level of Collagen Type XI Alpha 1 Chain fragment of collagen XVIII
Renal Cell Carcinoma	Type VII Type XXIII	the expression level of collagen type VII a 1 chain the expression of collagen type XXIII alpha 1 chain
Oral Cancer	Type IV	the expression of collagen type IV

**Table 3:** Cancer clinical trials of agents targeting collagen

Reference number	Phase	Drugs	Combination drugs	Cancer	Result
NCT05607017	0	Losartan	None	Breast Cancer	Recruiting
NCT01694589	0	LDE-225	None	Resectable Pancreatic Cancer	None
NCT00141297	I	PD-0332991	None	Advanced Cancer	Not certain
NCT01522989	I	PD-0332991	5-FU, Oxaliplatin	Advanced Solid Tumor Malignancies	None
NCT04106856	I	Losartan	Losartan Potassium	Resectable or Locally Advanced Pancreatic Cancer	Recruiting
NCT00303940	I	Talabostat Mesylate	Carboplatin, Temozolomide	Relapsed or Refractory Brain Tumors or Other Solid Tumors	None
NCT02889848	I	EN3835	None	Uterine Leiomyoma (Fibroids)	None
NCT01954355	I	LDE225	Paclitaxel	Advanced Solid Tumors	None
NCT02111187	I	LDE225	None	High-risk Localized Prostate Cancer	Not certain
NCT00053937	I	Pirfenidone	None	Neurofibromatosis Type I And Plexiform Neurofibroma	None
NCT00027677	I	Halofuginone Hydrobromide	None	Solid Progressive Tumor	None
NCT06725082	I	Recombinant Humanized Type III Collagen Injection	None	Breast Cancer	Recruiting
NCT00001683	I	COL-3	None	Refractory Metastatic Cancer	None
NCT05280873	I	Pirfenidone	Methylprednisolone	Checkpoint inhibitor-related pneumonitis	None
NCT06211335	IB	Losartan	None	Head and Neck Squamous Cell Carcinoma	Recruiting
NCT03900793	I/IB	Losartan	Sunitinib	Relapsed or Refractory Osteosarcoma	Recruiting
NCT03177291	I/IB	Pirfenidone	Carboplatin, Paclitaxel, Pemetrexed	Advanced Non-Small Cell Lung Cancer	None
NCT04467723	I/II	Pirfenidone	Atezolizumab	Stage IV And Recurrent Non- Small Cell Lung Cancer	Recruiting
NCT06484153	IB/II	Pirfenidone	Fruquintinib, Pembrolizumab	advanced or metastatic pMMR/MSS colorectal adenocarcinoma	Not yet recruiting
NCT04171219	II	Talabostat	None	Advanced Solid Cancer	None
NCT04439201	II	Palbociclib	None	Tumors With CCND1, 2, 3 Amplification	Not effective
NCT06135493	II	Losartan	None	Breast Cancer	Recruiting
NCT03951142	II	Losartan	None	Glioblastoma, Metastatic Brain Tumor	None
NCT05637216	II	Losartan	None	Breast Cancer	Recruiting
NCT00116389	II	Talabostat Mesylate	Gemcitabine	Stage IV Adenocarcinoma of the Pancreas	None
NCT00083239	II	Talabostat	None	Metastatic Melanoma	None
NCT00080080	II	Talabostat	Docetaxel	Advanced Non-Small Cell Lung Cancer	None
NCT00083252	II	Talabostat	Cisplatin	Advanced Melanoma	None
NCT00489710	II	Talabostat Mesylate	None	Metastatic Kidney Cancer	None
NCT00086203	II	Talabostat Mesylate	None	Advanced Chronic Lymphocytic Leukemia	None
NCT01613313	II	Collagenase Clostridium Histolyticum	None	Lipoma	None
NCT02249052	II	AA4500	None	Lipoma	Effective
NCT02002689	II	LDE225	None	PTCH1 or SMO Activated Solid and Hematologic Tumors	Not effective
NCT01327053	II	LDE225	None	Locally Advanced or Metastatic Basal Cell Carcinoma	Effective
NCT00754780	II	Pirfenidone	None	Neurofibromatosis Type I	None
NCT06142318	II	Pirfenidone	None	Head and Neck Squamous Cell Carcinoma	Recruiting
NCT00332033	II	Pirfenidone	None	Uterine Leiomyoma (Fibroids)	None
NCT00064142	II	Halofuginone Hydrobromide	None	HIV Related Kaposi’s Sarcoma	None
NCT04054245	II	LOXL2 Inhibitor PAT-1251	None	Primary Myelofibrosis, Post-Polycythemia Vera Myelofibrosis, or Post-Essential Thrombocytosis Myelofibrosis	None
NCT02195973	IIB	LDE225	None	Recurrent Platinum Resistant Ovarian Cancer	None
NCT00243204	III	Talabostat Mesylate	Docetaxel	Stage IIIB/IV Non-Small Cell Lung Cancer	None
NCT00290017	III	Talabostat	Pemetrexed	Advanced (Stage IIIB/IV) Non-Small Cell Lung Cancer	None

The above is from the ClinicalTrials.gov website

## Data Availability

No datasets were generated or analysed during the current study.

## Abbreviations

ICB: Immune checkpoint blockade
ECM: Extracellular matrix
CFAs: Cancer-associated fibroblasts
CI cycle: Cancer-immunity cycle
COL I: Collagen type I
COL III: Collagen type III
COL V: Collagen type V
COL IV: Collagen type IV
Col XVII: Collagen type XVII
COL XVIII: Collagen type XVIII
VEC: Vascular endothelial cell
EMT: Epithelial–mesenchymal transition
DDRs: Discoidin domain receptors
RER: Rough endoplasmic reticulum
MMPs: Matrix metalloproteinases
FAK: Focal adhesion kinase
NK cell: Natural killer cell
Tregs: Regulatory T cells
TAMs: Tumor-associated macrophages
CSF1: colony stimulating factor 1
IL-6: Interleukin-6
OV: Ovarian serous cystadenocarcinoma
DCs: Dendritic cells
CTLs: Cytotoxic T cells
ICIs: Immune checkpoint inhibitors
CAR-T: Chimeric antigen receptor T
Asbs-HC: Hydrolyzed collagen from defatted Asian sea bass
EECs: Endocardial endothelial cells
PKC: Protein kinase C
MAPK: Mitogen-activated protein kinases
TECs: Tumor endothelial cells
NSCLC: Non–small cell lung cancer
HCC: Hepatocellular carcinoma
HIF-1: Hypoxia-inducible factor 1
AFP: Alpha-fetoprotein
CEA: Carcinoembryonic antigen
PSA: Prostate-specific antigen
CA-19-9: carbohydrate antigen 19-9
cCA15-3: circulating CA15-3
MBC: Metastatic breast cancer
CRC: Colorectal cancer
GC: Gastric cancer
PDAC: Pancreatic ductal adenocarcinoma
RCC: Renal cell carcinoma
SCLC: Small cell lung cancer
PCa: Prostate cancer
CBD: Collagen-binding domain
TCRs: T-cell receptors
TME: Tumor microenvironment
PRO-C3: Propeptide of type III collagen
irAEs: Immune-related adverse events
CBP: Collagen-binding protein
